# Investigation of the Hot Stamping-in-Die Quenching Composite Forming Process of 5083 Aluminum Alloy Skin

**DOI:** 10.3390/ma16072742

**Published:** 2023-03-29

**Authors:** Lingling Yi, Ge Yu, Ziming Tang, Xin Li, Zhengwei Gu

**Affiliations:** 1 Department of Materials Science and Engineering, Jilin University, Changchun 130022, China; 2 State Key Laboratory of Automobile Materials, Jilin University, Changchun 130022, China; 3 Roll Forging Research Institute, Jilin University, Changchun 130022, China

**Keywords:** Al5083, hot stamping, finite element method, high-speed train skin

## Abstract

Aluminum alloy has been used as the skin material for rail vehicles and automobiles to meet the requirements of environmental protection. The hot stamping-in-die quenching composite forming (HFQ) process is a promising technology to compensate for the poor formability of the aluminum alloy sheet at room temperature. In this paper, the high-temperature mechanical properties of 5083 aluminum alloy under various temperature (200 °C, 300 °C, 400 °C, 450 °C) and strain rate conditions (0.01 s^−1^, 0.10 s^−1^, 1.00 s^−1^) were investigated by uniaxial tensile tests. The finite element software of PAM-STAMP was employed to simulate the forming process of high-speed train skin. The effects of forming method and process parameters on the minimum thickness and springback of the skin were analyzed using the Response Surface Methodology (RSM). After parameter optimization, the forming experiment verified the simulation results and the test part met the quality requirements: the thickness above 3.84 mm and the springback within 1.1 mm. Mechanical properties of the sheet before and after HFQ were examined by uniaxial tensile tests at room temperature. It can be inferred from the comparison that the yield strength of the Al5083 sheet increases, but the elongation decreases from the HFQ process.

## 1. Introduction

The problems of environmental degradation and energy shortage are worsening with the continuous development of industry and transportation. The development and application of lightweight technology are indispensable in achieving the goals of energy saving and emissions reduction [1]. Light weight products are often achieved by optimizing the structure and using lightweight materials [2,3]. Compared with steel, aluminum alloys have the advantages of low density and high specific strength and are widely used in the field of lightweight materials [4,5,6]. In the aviation industry, aluminum alloys are mainly employed in components such as fuselage skins, wings, pressure chambers, and fairing [7,8,9]. Additionally, there is a trend for aluminum to be used as a body material [10,11]. The greenhouse gas emissions of passenger cars using aluminum alloys are lower than those of regular passenger cars from a life-cycle perspective [12]. Furthermore, aluminum alloy is a predominant material used in the body of high-speed trains [13,14,15]. Deformed aluminum alloys are divided into two types according to whether or not they can be strengthened by heat treatment. The 2xxx and 7xxx aluminum alloys are granted improved strength primarily by heat treatment operations to modulate the diffusely precipitated second phase [16,17]. The 5xxx aluminum alloy differs from them because the transition phase produced during aging is not coherent with the matrix, and the equilibrium phase is prone to distribution along grain boundaries [18]. This type of alloy is usually used only in the annealed or cold-hardened state.

Cold stamping represents the prevalent forming method for aluminum alloy sheets owing to its high production efficiency and low cost. The poor forming performance at room temperature is prone to defects, such as cracking and springback in the cold stamping process [19,20], which limits the use of aluminum alloys. Lin et al. [11] examined the formability of stamping an engine hood with an A6181-T4 aluminum alloy sheet and changed the stamping process parameters to eliminate the crack defect in the test part. Huang et al. [21] studied the effect of different yield functions on the springback prediction of automotive parts stamped from the 5754 aluminum alloy sheet.

The researchers have focused on the deformation behavior of aluminum alloys at elevated temperatures to overcome the drawback [22,23]. The superplastic forming (SPF) process is applied to form parts with high accuracy. However, low productivity and non-uniform thickness distribution are its major problems [24]. A new technology called the quick superplastic forming process combines the hot drawing pre-forming and superplastic forming processes. Some researchers have applied this manufacturing process to make the side wall outer panel of metro vehicles and bonnets [25,26]. The hot stamping-in-die quenching composite forming process (HFQ) for aluminum alloys was proposed by Lin [27,28,29,30,31,32]. This process is an ideal method for solving the problems of aluminum alloys at room temperature and is more productive than SPF [33,34,35,36]. Fan et al. [37] investigated the HFQ process of 6A02 aluminum alloy sheets at different forming-die temperatures ranging from 50 °C to 350 °C and found the temperature should not be higher than 250 °C to obtain a sufficient strengthening effect. Harrison [16] used the method of hot stamping and then artificial aging to form the 7075 aluminum alloy B-pillar outer plate with little springback. Wang et al. [38] tested the ductility of AA2024 at different temperatures. It has very high formability at around 450 °C, as confirmed by the cup test.

In this paper, the HFQ process was used to form a large-size aluminum alloy high-speed train skin. The effect of the two processes (hot stamping and drawing) on the forming quality of the skin was compared by finite element methods. Response Surface Methodology (RSM) was adopted to explore the effect of parameters including the stamping speed, die clearance, and holding time on the minimum thickness and springback, and to optimize and obtain the appropriate process parameters. The forming experiment was performed using the simulation results to verify the accuracy of the FEA. Uniaxial tensile tests have verified the strengthening effect of this process on Al5083.

## 2. Material and Methods

### 2.1. Material 

The material of the high-speed train skin is Al5083 in H111 condition with a thickness of 4 mm. The main chemical composition is shown in Table 1.

**Figure 1:**
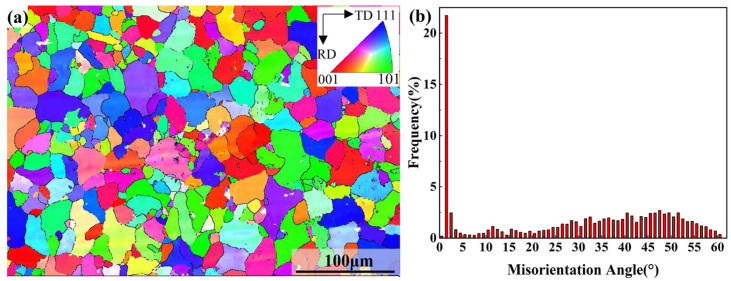
Initial microstructure of Al5083: (**a**) EBSD IPF map. (**b**) Misorientation angle distribution.

Electron Backscattered Diffraction (EBSD) detection was performed on the RD-TD surface of the specimen. The initial microstructure of Al5083 is shown in Figure 1. Figure 1a was based on the inverse pole figure (IPF) of (001) and was color coded according to its grain orientation. It can be seen that Al5083 is composed of the equiaxed grains with an average grain size of 12.87 µm. Figure 1b shows the distribution of misorientation angle. The misorientation angle is defined as lower than 15° as low-angle grain boundaries (LAGBs), and higher than 15° as high-angle grain boundaries (HAGBs). It presented with 70% as the HAGBs fraction and 30% as the LAGBs fraction, which indicates that the material exhibits good toughness.

### 2.2. Material Model

**Figure 2:**
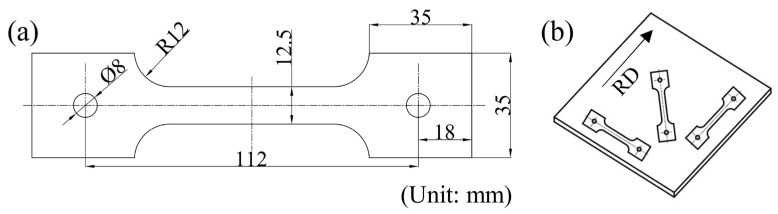
(**a**) The size of the hot uniaxial tensile specimen; (**b**) Cutting scheme of the specimen.

To investigate the mechanical properties of Al5083 at high temperature, the hot uniaxial tensile tests were performed in a WDW-300 electronic universal testing machine. The size of the tensile specimen is shown in Figure 1. Forces and displacements were automatically collected during the tensioning process. The PID thermostat automatically controlled the temperature with an accuracy of ±2 °C in the heating process. At present, there are many studies on the hot forming process of aluminum alloys, and the deformation temperature ranges basically between 200 °C and 450 °C [39,40,41]. Moreover, the strain rate of the sheet is high during hot stamping and the deformation mechanism is a glide-controlled thermally activated dislocation mechanism, instead of the Grain Boundary Sliding (GBS) and the Solute Drag creep (SD) that prevail in SPF and Quick-Plastic Forming (QPF), respectively [42]. Therefore, the tensile tests were conducted at different temperatures of 200 °C, 300 °C, 400 °C, and 450 °C. The strain rates at each temperature were 0.01 s^−1^, 0.1 s^−1^, and 1 s^−1^. To investigate the anisotropy of the material under these temperatures and strain rate conditions, the tensile specimens were cut in directions that are 0°, 45°, and 90° from the rolling direction (RD), as shown in Figure 2b.

**Figure 3:**
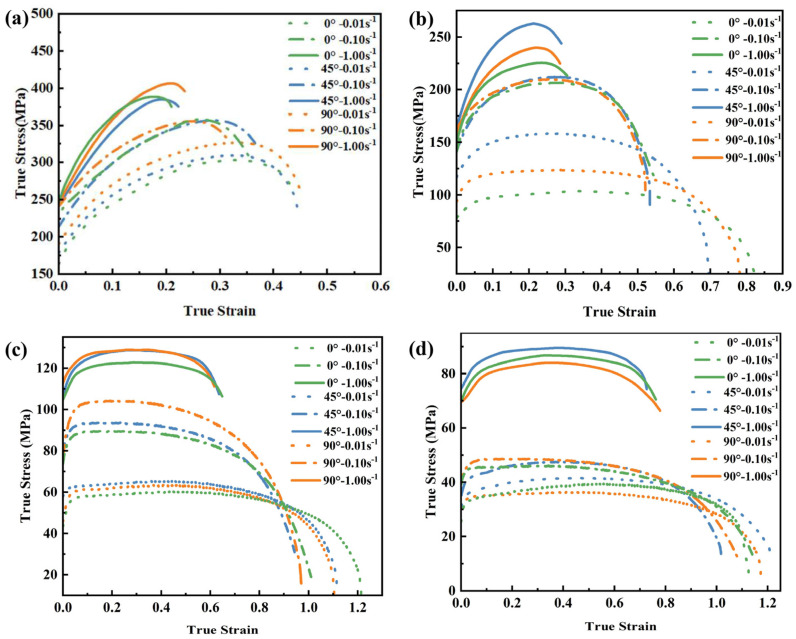
The true stress–true plastic strain curves for Al5083 at different temperatures: (**a**) 200 °C; (**b**) 300 °C; (**c**) 400 °C; (**d**) 450 °C.

The true stress–true plastic strain curves for Al5083 at different temperatures are shown in Figure 3. It is clearly seen that increasing the strain rate or decreasing the deformation temperature results in a monotonic increase in the flow stress before reaching the tensile strength. At the same temperature, elongation rises as the strain rate decreases. Furthermore, the higher the temperature, the greater the elongation with the same strain rate. The elongation exceeds 230% when the specimen was subjected to tensile stress at 450 °C with a strain rate of 0.01 s^−1^. Because the larger strain rate leads to a higher dislocation density, the dynamic recovery process cannot reduce the dislocation density effectively within the limited deformation time. Moreover, the deformation at low-temperatures leads to more stored energy, which increases the driving force for grain boundary sliding but also reduces the ability of recovery and grain boundary sliding. It can also be found that under the same deformation conditions, the higher the temperature, the smaller the strain rate and the greater the difference in mechanical properties caused by the tensile direction. These curves were input to PAM-STAMP as discrete data points. The mechanical properties of the material at other temperatures and strain rate conditions during forming would be calculated by the interpolation. The yield function of the Hill48 model considering only the normal anisotropy [43] is expressed as follows:

(1) $$\sigma_{e}{}^{2}{=\sigma }_{1}{}^{2}-\frac{2R}{1+R}\sigma_{1}\sigma_{2}{+\sigma }_{2}{}^{2}$$

where R is the normal anisotropy index, which is strongly influenced by temperature and strain rate. Therefore, for convenient calculation and safe engineering design, and the average normal anisotropy index R was set to 0.7 [44].

**Figure 4:**
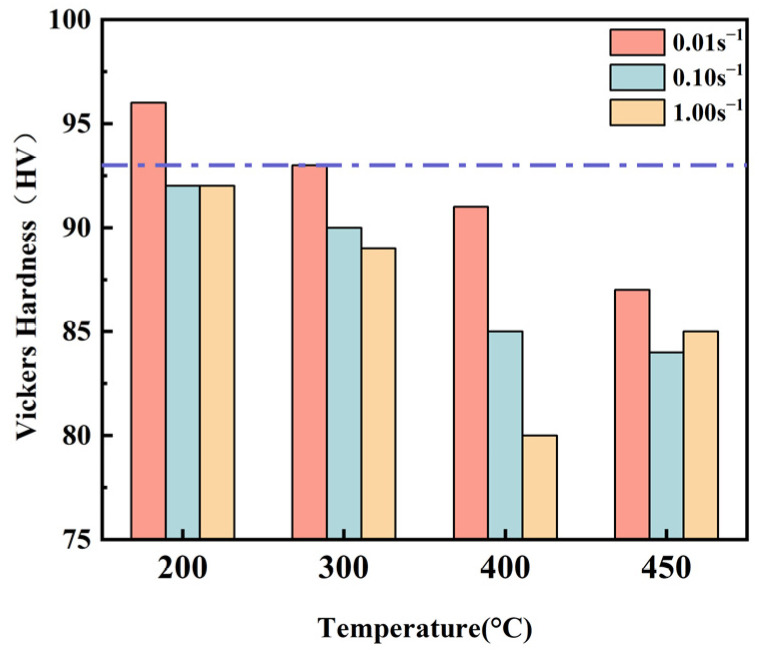
Vickers hardness at different temperatures and strain rates for the Al5083.

Micro-hardness tests were performed on specimens that had undergone tensile tests, and Figure 4 shows the Vickers hardness of the Al5083 at different temperatures and strain rates. The Vickers hardness initial sheet is 93 HV. The Vickers hardness basically decreases with increasing temperature and strain rate. Overall, the Vickers hardness shows little change compared to the as-received state. 

### 2.3. Finite Element Modeling of the HFQ Process

**Figure 5:**
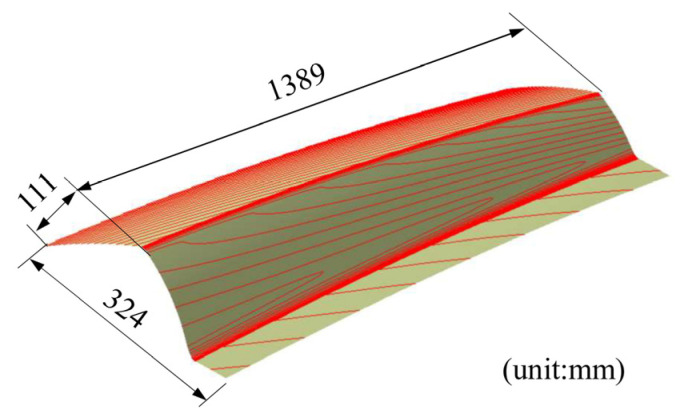
The geometric model of the aluminum alloy skin.

The geometric model of the aluminum alloy skin is shown in Figure 5. The part is large and there are some complex space shapes with local sharp edges and corners. It is difficult to form a part of high quality using cold forming because the sharp corner seems to be at risk of cracking and the springback may lead to poor shape accuracy.

In the process of numerical simulation, a commercial finite element software PAM-STAMP was developed with the coupled temperature–displacement deformation mode. The tools and the blank were discretized using Belytschko–Tsay (BT) shell elements. The initial size of the mesh was 5 mm, and the maximum adaptive level was 4 to guarantee both calculation speed and accuracy. The Coulomb friction model was used in the hot stamping process with a friction coefficient of 0.13.

**Figure 6:**
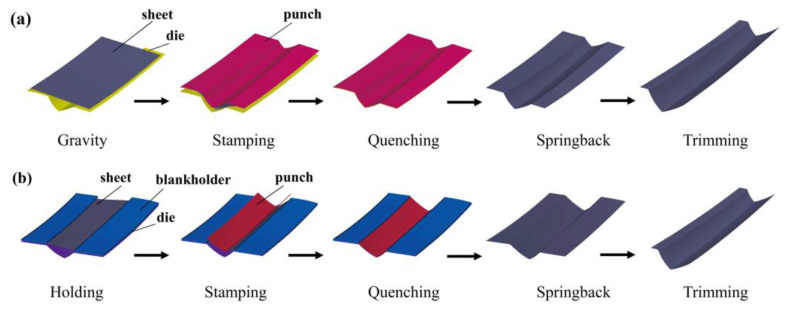
FE simulation procedures for (**a**) hot stamping and (**b**) drawing.

In order to obtain the die surface, the part model was supplemented with a process that guarantees the same curvature of the part. The part can be produced by either the hot stamping or the drawing process. The FE simulation procedures for the two processes are shown in Figure 6. The hot stamping process includes gravity loading, stamping, quenching, springback, and trimming. When the hot stamping process is adopted, the sheet would wrinkle or show springback deformation because it is difficult to control the sheet’s flow. Drawing has an additional step of holding before stamping compared to the hot stamping process. The sheet would crack because of the low flow stress at high temperature if the drawing process is employed. Therefore, it is necessary to discuss the effect of the two forming processes on formability.

## 3. Results and Discussion

### 3.1. Effect of the Forming Process on the Forming Result

**Figure 7:**
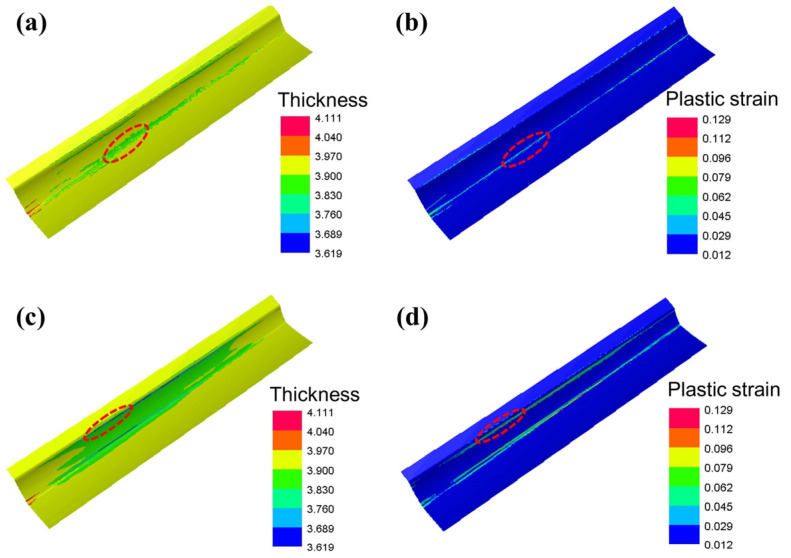
The thickness and plastic strain distribution of the skin formed by the two processes: (**a**,**b**) hot stamping; (**c**,**d**) drawing. The area circled by the red origin is the area with the most severe thinning.

The thickness and plastic strain distribution of the skin formed by the two processes are shown in Figure 7. As seen in Figure 7a, the thickness of the sheet at the sharp central corner is thinnest, where the equivalent strain is 0.055 when the stamping is completed (Figure 7b). However, the minimum thickness of the skin formed by drawing is 3.619 mm, located at the entrance fillet of the die as the effect of the blankholder (Figure 7c). From Figure 7d, it can be seen that the equivalent plastic strain at the corresponding location is 0.104. Moreover, the thickness distribution of the skin formed by hot stamping is more uniform than another.

**Figure 8:**
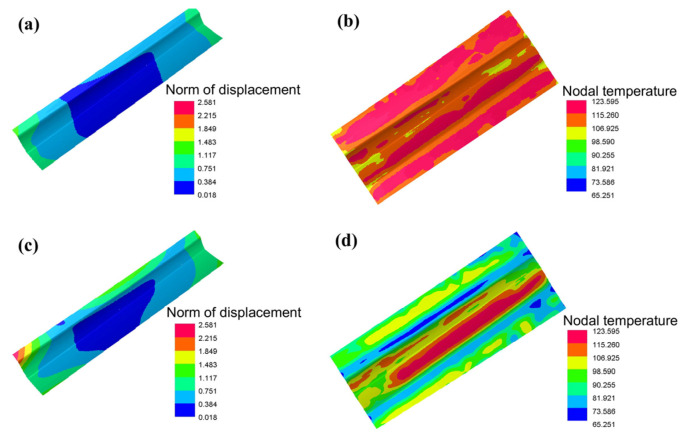
The springback and temperature distribution of the skin formed by the two processes: (**a**,**b**) Hot stamping. (**c**,**d**) Drawing.

Springback is an important indicator to judge the quality of the part. The greater the springback, the worse the shape accuracy. The springback distribution of the skin formed by the two processes is shown in Figure 8a,c. The springback increases gradually from the middle to the two edges because it is hard for the center of the sheet to deform owing to the inhibition of the free end sheet. When using the drawing process, the maximum springback is 2.581 mm, as shown in Figure 8c. The reason can be analyzed by looking at the temperature distribution of the sheet before removing it from the die (Figure 8b,d). The greater the difference in temperature between the pressing and the deformed area, the larger the deformation by releasing thermal stress.

**Figure 9:**
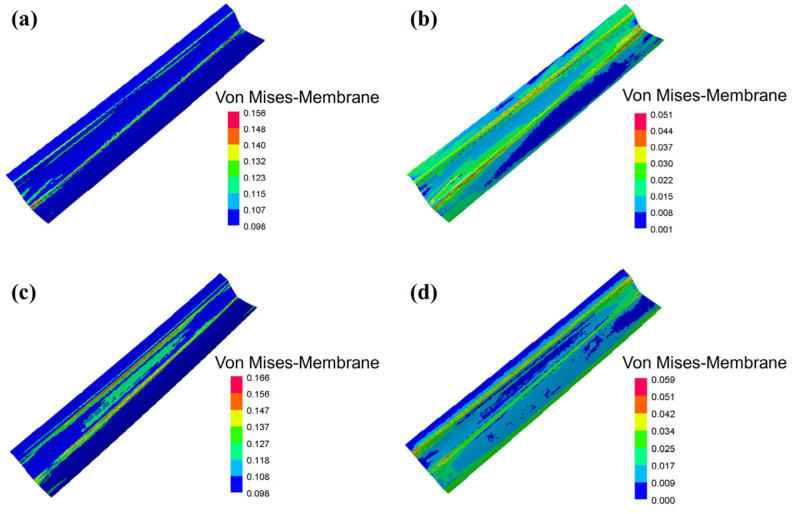
von Mises stress distribution before and after springback: (**a**,**b**) Hot stamping.; (**c**,**d**) Drawing.

To further illustrate the reason for the springback distribution, the von Mises stress distribution of the blank before and after springback were analyzed for the two processes, as shown in Figure 9. Springback is a stress-release process, thus the von Mises stress is much lower after springback. The maximum stress in the sheet formed by hot stamping before springback is 0.166 GPa (Figure 9c), which is 0.01 GPa greater than that of the sheet formed by drawing (Figure 9a). The hot stamping process causes a smaller springback.

Considering the thinning and springback, it is more suitable for the high-speed train skin to form by hot stamping.

### 3.2. Effect of Forming Parameters on the Forming Result

In hot stamping, the stamping speed affects the sheet temperature during deformation. Thus, it directly changes the deformation capacity of the material. The die clearance is closely related to the shape accuracy of the part and the wear of the die. The springback is strongly influenced by the holding time. The minimum thickness reflects uniformity, and the springback represents the shape accuracy of the workpiece. The Response Surface Methodology (RSM) could derive a continuous functional relationship between the responses and multiple factors [45]. Therefore, RSM was adopted to explore the effect of parameters including the stamping speed, die clearance, and holding time, on the minimum thickness and springback, and to optimize and obtain the appropriate process parameters. The experimental design was performed using the Box–Behnken Design (BBD) in order to avoid too many experiments reducing the computational efficiency. The experimental design and results are presented in Table 2.

**Figure 10:**
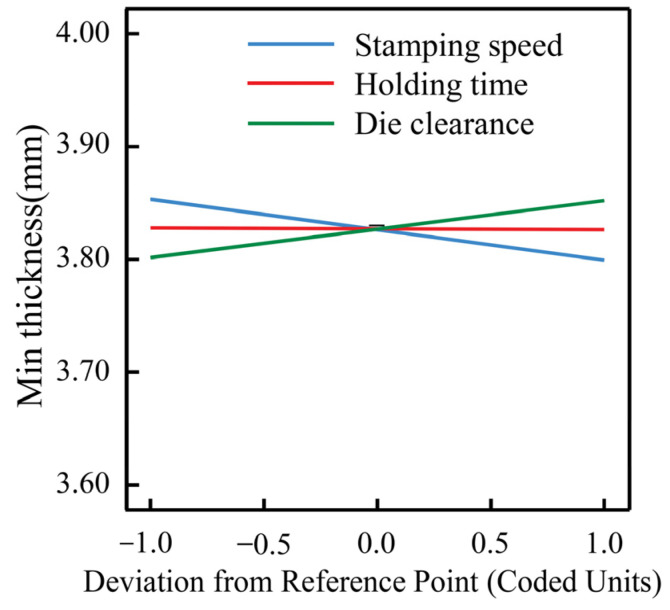
The effect of the single factor on the thickness.

A linear model was established between the minimum thickness and the process parameters after analysis, as shown in Equation (2). The model F-value of 7.4 implies the model is significant. The functional relationship diagram is shown in Figure 10. In this experimental scheme, the minimum thickness varies in a small range, from 3.717 mm to 3.865 mm, which meets thinning rate requirements of less than 10%. The minimum thickness is inversely correlated with the die clearance and directly correlated with the stamping speed. When the stamping speed becomes faster, the more serious the local plastic deformation and the more obvious the thinning. The die clearance is related to the deformation of the sheet, with the increase in the die clearance, the smaller the deformation. However, the effect of holding time on the minimum thickness is negligible.

R1 = 3.83 − 0.027A + 0.0251B − 0.0004C(2)

**Figure 11:**
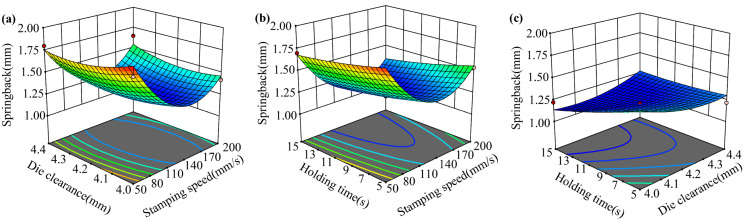
The effect of the two-factor interaction on the springback: (**a**) A–B; (**b**) A–C; (**c**) B–C.

A quadratic model with an F-value of 14.08 was developed between the springback and the process parameters, as shown in Equation (3). The influence of parameters on springback is complex and the interaction between the factors needs to be considered. The effect of the two-factor interaction on the springback is shown in Figure 11. The springback is related to the temperature distribution of the sheet at the end of the hot stamping. If the temperature of the sheet is low and evenly distributed before opening the mold, then the springback will be smaller.

R2 = 41.345 − 0.033A − 17.051B − 0.379C + 0.004AB − 0.00003AC + 0.0828BC + 0.00007A^2^ + 1.872B^2^ + 0.0008C^2^ (3)

### 3.3. Optimization of the Process Parameters

In order to obtain the part that meets the thickness and shape requirements, the process parameters were optimized using the hill climbing technique based on the established regression model. To maximize the minimum thickness and minimize the springback, the optimized parameter combination was stamping speed of 131 mm/s, die clearance of 4.16 mm, and holding time of 15 s.

## 4. Experimental Verification

**Figure 12:**
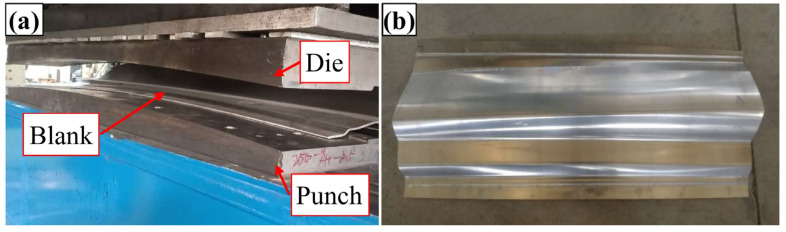
(**a**) The tools for the forming experiment; (**b**) test part.

The forming experiment was conducted by a box-type resistance furnace with a big chamber size of 2500 × 2000 × 1000 mm and a maximum heating temperature of 960 °C. The sheet was formed at 450 °C since the plasticity was greatest at this temperature [46]. The tools were made of H13 steel with high hardenability and resistance to thermal cracking (Figure 12a). When machining tools, a clearance of 4.16 mm was kept between the punch and the die. The friction between the sheet and the tool has a significant impact on the service life of the tool and the forming quality of the part [47,48]. Boron nitride is a common lubricant employed in aluminum alloy hot stamping, and its Coulomb friction coefficient under this condition is 1–1.5 [49]. The boron nitride was uniformly sprayed on both sides of the sheet before the experiment. The sheet was heated to 480 °C in the heating furnace and then kept there for 2 min. Then, the sheet was transferred to the die with the heat insulation clamp, and when the temperature dropped to 450 °C, the punch was moved down at a speed of 131 mm/s. Holding pressure lasted for 15 s after closing the die, and then the sheet was air-cooled to room temperature; the test part is shown in Figure 12b.

### 4.1. Thickness and Shape Checking

**Figure 13:**
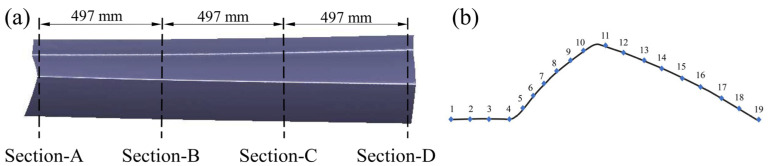
(**a**) Thickness detection sections. (**b**) The positions of the measurement points in each section.

**Figure 14:**
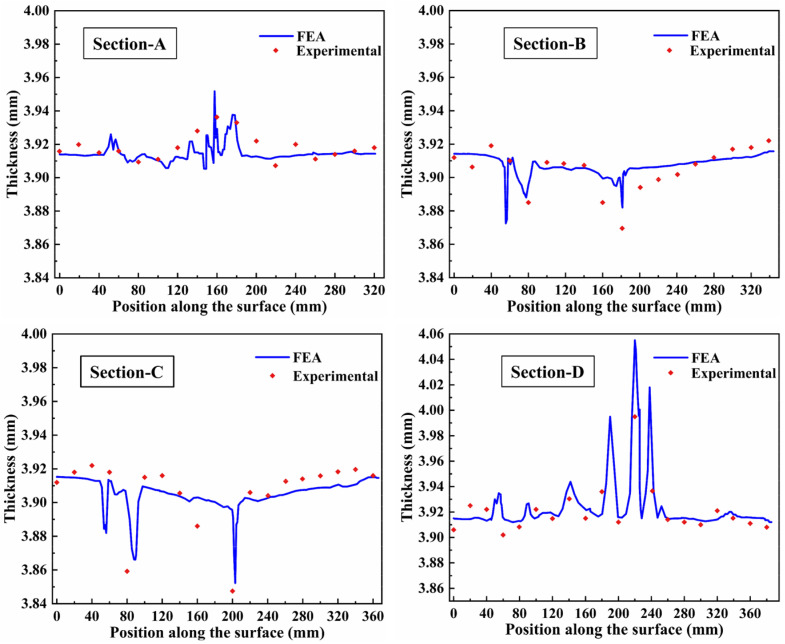
Comparison between simulation and experimental thickness distribution of the skin in each section.

As shown in Figure 13a, the test part was cut at 497 mm intervals and the cutting sections were defined as Sections A–D. Each section was divided into 18 segments with 19 separation points, and the thickness at these points was measured (Figure 13b). A comparison between numerical and experimental thickness distributions of the skin in four sections is shown in Figure 14. Section-A and Section-D are located at the ends of the skin and show slight wrinkling at the bottom sharp corner and entrance fillet, for the shallow forming depth. However, sections B and D are located in the middle of the skin, and the fillets thinning obviously caused by the large deformation. It can be seen that the deviation of thickness for the four sections is less than 5% from the experimental result. The finite element model correctly predicted the quality of the part formed using the HFQ process. The thickness of the skin was uniformly distributed (the range is 3.86–4.00 mm). The max thinning was less than 10%, which meets the technical requirements.

**Figure 15:**
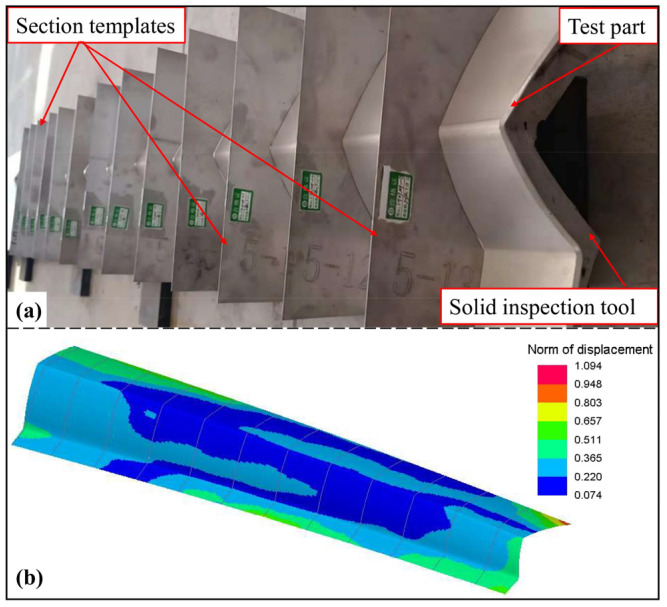
(**a**) Test part shape accuracy detection. (**b**) The springback of the numerical result.

The shape accuracy of the outer surface is important for the skin, in order to ensure the beauty of the high-speed train. The skin was placed on the solid inspection tool to be positioned. As shown in the Figure 15a, the section templates are machined by selecting 13 positions along its longitudinal direction using the laser cutting method, based on the shape of the outer surface of the standard part. The clearance between the test part and the template was measured using a feeler gauge and was defined as the section accuracy deviation. It can be seen from Figure 15b that the accuracy deviations of the 13 sections meet the accuracy requirement of less than 1.1 mm and are nearly consistent with the simulation results.

### 4.2. Material Properties Checking

**Figure 16:**
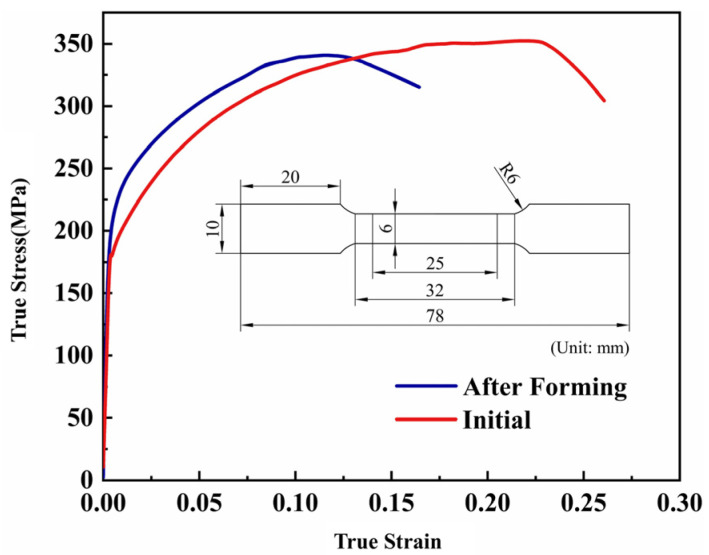
The geometry of the specimen for tensile tests and true stress–true strain curve of Al5083.

In order to test the material properties of the parts, a tensile test was performed. The geometry of the specimen for the tensile tests and the true stress–true strain curves of the initial and deformed sheet at room temperature are shown in Figure 16. After deformation, the yield strength increased by 18.5% and the elongation after fracture decreased significantly because of work-hardening of the material. The dislocation density increased as plastic deformation proceeded, resulting in dislocation entanglement and other obstacles. Therefore, E in dislocation resistance allows the strength of the alloy to be higher.

## 5. Conclusions

In this paper, the HFQ process of Al5083 high-speed train skin was investigated by finite element simulation and forming experiments. The conclusions are as follows:

1. The tensile tests of Al5083 in directions that are 0°, 45°, and 90° from RD at different temperatures (200 °C, 300 °C, 400 °C, 450 °C) and strain rates (0.01 s^−1^, 0.10 s^−1^, 1.00 s^−1^) revealed that increasing the strain rate or decreasing the deformation temperature resulted in a monotonic increase in the flow stress. The elongation increased with the decreasing strain rate at the same temperature. For the same strain rate, the higher the temperature, the higher the elongation. Elongation could exceed 230% when the specimen was tensile at 450 °C with a strain rate of 0.01 s^−1^.
2. The hot stamping process was proven by finite element simulation to be more suitable for the formation of skin than the hot drawing at 450 °C. The effects of stamping speed, die clearance, and holding time on forming quality during the HFQ process were investigated. Based on the RSM, the linear and quadratic regression models were developed for the process parameters related to minimum thickness and springback, respectively. The optimized process parameters for forming the skin were stamping speed of 131 mm/s, die clearance of 4.16 mm, and a holding time of 15 s.
3. Checking the thickness and shape of the skin proved the exactness of the numerical simulation. The minimum thickness was larger than 3.84 mm and the springback was less than 1.1 mm, which meet the quality requirements of the part.
4. Conducting uniaxial tensile tests on the initial and deformed sheets at room temperature verified that the HFQ process had a positive influence on the mechanical properties, and the yield strength was increased by 18.5%.

## Figures and Tables

**Table 1:** Chemical composition of Al5083 (wt.%).

Mg	Si	Fe	Cu	Mn	Zn	Cr	Ti	Al
4.30	0.40	0.30	0.10	0.60	0.25	0.20	0.15	rest

**Table 2:** Experimental design and results.

Run	A	B	C	R1	R2
	Stamping Speed (mm/s)	Die Clearance (mm)	Holding Time (s)	Min Thickness (mm)	Springback (mm)
1	200	4.2	5	3.8	1.54
2	50	4.2	15	3.844	1.7
3	125	4.2	10	3.838	1.21
4	200	4.4	10	3.85	1.65
5	200	4.0	10	3.717	1.42
6	50	4.0	10	3.842	1.78
7	125	4.2	10	3.838	1.21
8	125	4.2	10	3.838	1.21
9	125	4.0	5	3.82	1.56
10	125	4.2	10	3.838	1.21
11	125	4.0	15	3.815	1.224
12	50	4.2	5	3.832	1.89
13	200	4.2	15	3.800	1.3
14	50	4.4	10	3.865	1.8
15	125	4.4	5	3.845	1.22
16	125	4.2	10	3.838	1.21
17	125	4.4	15	3.865	1.215

## Data Availability

Datasets generated and/or analyzed during the current study are available from the corresponding author on request.

## References

[1] Buljac A., Hild F., Helfen L., Morgeneyer T.F. (2018). On deformation and damage micromechanisms in strong work hardening 2198 T3 aluminium alloy. Acta Mater..

[2] Zhu L.J., Liu Z.X., Zhang Z.Q. (2019). Investigation on strengthening of 7075 aluminum alloy sheet in a new hot stamping process with pre-cooling. Int. J. Adv. Manuf. Technol..

[3] Hirsch J., Al-Samman T. (2013). Superior light metals by texture engineering: Optimized aluminum and magnesium alloys for automotive applications. Acta Mater..

[4] Lutsey N. (2010). Review of Technical Literature and Trends Related to Automobile Mass-Reduction Technology.

[5] Chen G.L., Chen M.H., Wang N., Sun J.W. (2016). Hot forming process with synchronous cooling for AA2024 aluminum alloy and its application. Int. J. Adv. Manuf. Technol..

[6] Fan X., He Z., Yuan S., Lin P. (2013). Investigation on strengthening of 6A02 aluminum alloy sheet in hot forming-quenching integrated process with warm forming-dies. Mater. Sci. Eng. A.

[7] Schuster P., Österreicher J., Kirov G., Sommitsch C., Kessler O., Mukeli E. (2019). Characterisation and Comparison of Process Chains for Producing Automotive Structural Parts from 7xxx Aluminium Sheets. Metals.

[8] Barnes A.J., Raman H., Lowerson A., Edwards D. (2012). Recent Application of Superformed 5083 Aluminum Alloy in the Aerospace Industry. Mater. Sci. Forum.

[9] Dursun T., Soutis C. (2014). Recent developments in advanced aircraft aluminium alloys. Mater Des..

[10] Gullino A., Matteis P., D’Aiuto F. (2019). Review of Aluminum-To-Steel Welding Technologies for Car-Body Applications. Metals.

[11] Friedman P.A., Luckey S.G., Copple W.B., Allor R., Miller C.E., Young C. (2004). Overview of Superplastic Forming Research at Ford Motor Company. J. Mater. Eng. Perform..

[12] Lin C.W., Chen F.K. (2019). Formability study on stamping an engine hood with aluminum alloy sheet. IOP Conf. Ser.-Mater. Sci. Eng..

[13] Modaresi R., Pauliuk S., Lovik A.N., Muller D.B. (2014). Global Carbon Benefits of Material Substitution in Passenger Cars until 2050 and the Impact on the Steel and Aluminum Industries. Environ. Sci. Technol..

[14] Huang S.H., Xu Y.G., Bezold A., Zhang L.L., Chen G., Broeckmann C. (2019). A direct method-based strength evaluation of the cast aluminum beam used in a high-speed train. Proc. Inst. Mech. Eng. Part F J. Rail Rapid Transit.

[15] Lu W., Ma C.P., Gou G.Q., Fu Z.H., Sun W.G., Che X.L., Chen H., Gao W. (2021). Corrosion fatigue crack propagation behavior of A7N01P-T4 aluminum alloy welded joints from high-speed train underframe after 1.8 million km operation. Mater. Corros..

[16] Zhao G.Q., Chen H., Zhang C.S., Guan Y.J., Gao A.J., Peng L. (2014). Die optimization design and experimental study of a large wallboard aluminum alloy profile used for high-speed train. Int. J. Adv. Manuf. Technol..

[17] Harrison N.R., Luckey S.G. (2014). Hot Stamping of a B-Pillar Outer from High Strength Aluminum Sheet AA7075. SAE Int. J. Mater. Manuf..

[18] Yarar E., Erturk A.T., Karabay S. (2021). Dynamic Finite Element Analysis on Single Impact Plastic Deformation Behavior Induced by SMAT E in 7075-T6 Aluminum Alloy. Met. Mater. Int..

[19] Ertürk A.T., Şahin M., Aras M. (2017). Tribological Behavior of SiC Particulate Reinforced AA5754 Matrix Composite Under Dry and Lubricated Conditions. Trans. Indian Inst. Met..

[20] Kumar M., Ross N.G. (2017). Investigations on the Hot Stamping of AW-7921-T4 Alloy Sheet. Adv. Mater. Sci. Eng..

[21] Wang X.Y., Li J.B., Deng L., Li J.J. (2018). Metal flow control during hot forming of square cups with local-thickened plates and varied friction conditions. J. Mater. Process. Technol..

[22] Huang M.D., Fu L., Lee L., Liu C. (2018). Effect of Yield Function on the Stamping Springback of Aluminum Alloy. High Performance Structural Materials, Proceedings of the Chinese Materials Conference 2017, Yinchuan, China, 6–12 July 2017.

[23] Liang J.C., Gao S., Teng F., Yu P.Z., Song X.J. (2014). Flexible 3D stretch-bending technology for aluminum profile. Int. J. Adv. Manuf. Technol..

[24] Palumbo G., Tricarico L. (2007). Numerical and experimental investigations on the Warm Deep Drawing process of circular aluminum alloy specimens. J. Mater. Process. Technol..

[25] Balasubramanian M., Ganesh P., Ramanathan K., Senthil Kumar V.S. (2015). Superplastic Forming of a Three-Stage Hemispherical 5083 Aluminium Profile. Stroj. Vestn.-J. Mech. Eng..

[26] Liang H.J., Wu X.W., Wang Y., Jin Q.L., Ma Z.L., Feng S.S. (2012). Research on Quick Superplastic Forming for Aluminium Alloy Sheet. Mater. Sci. Forum.

[27] Guofeng W., Chao S., Shufen L., Mo Y. (2014). Research on quick superplastic forming technology of aluminum alloy complex components. Mater. Werkst..

[28] Shao Z.T., Jiang J., Lin J.G. (2018). Feasibility study on direct flame impingement heating applied for the solution heat treatment, forming and cold die quenching technique. J. Manuf. Process..

[29] Shao Z.T., Lee J., Wang J.L., Lin J.G., Jiang J. (2020). A study of various heating effects on the microstructure and mechanical properties of AA6082 using EBSD and CPFE. J. Alloys Compd..

[30] Wang L.L., Dean T., Lin J.G. (2017). Innovation, Development and Implementation of the HFQ (R) Process. Proceedings of the 3rd International Conference on Advanced High Strength Steel and Press Hardening (ICHSU2016), Xi’an, China, 25–27 August 2016.

[31] Zheng K.L., Dong Y.C., Zheng D.Q., Lin J.G., Dean T.A. (2019). An experimental investigation on the deformation and post-formed strength of heat-treatable aluminium alloys using different elevated temperature forming processes. J. Mater. Process. Technol..

[32] Zheng K.L., Dong Y.C., Zheng J.H., Foster A., Lin J.G., Dong H.S., Dean T.A. (2019). The effect of hot form quench (HFQ (R)) conditions on precipitation and mechanical properties of aluminium alloys. Mater. Sci. Eng. A-Struct..

[33] Zheng K.L., Zhu L., Lin J.G., Dean T.A., Li N. (2019). An experimental investigation of the drawability of AA6082 sheet under different elevated temperature forming processes. J. Mater. Process. Technol..

[34] Wang X., Zhou G., Men Y., Zhang S., Zhang H., Li F., Chen L. (2022). Superplastic Deformation Behaviors and Power Dissipation Rate for Fine-Grained Ti-6Al-4V Titanium Alloy Processed by Direct Rolling. Crystals.

[35] Sun P.-H., Wu H.-Y., Lee W.-S., Shis S.-H., Perng J.-Y., Lee S. (2009). Cavitation behavior in superplastic 5083 Al alloy during multiaxial gas blow forming with lubrication. Int. J. Mach. Tools Manuf..

[36] Xu Y., Lv X.W., Wang Y., Zhang S.H., Xie W.L., Xia L.L., Chen S.F. (2023). Effect of Hot Metal Gas Forming Process on Formability and Microstructure of 6063 Aluminum Alloy Double Wave Tube. Materials.

[37] Mikhaylovskaya A.V., Yakovtseva O.A., Irzhak A.V. (2022). The role of grain boundary sliding and intragranular deformation mechanisms for a steady stage of superplastic flow for Al–Mg-based alloys. Mater. Sci. Eng. A.

[38] Fan X., He Z., Yuan S., Zheng K. (2013). Experimental investigation on hot forming–quenching integrated process of 6A02 aluminum alloy sheet. Mater. Sci. Eng. A.

[39] Wang L., Strangwood M., Balint D., Lin J., Dean T.A. (2011). Formability and failure mechanisms of AA2024 under hot forming conditions. Mater. Sci. Eng. A.

[40] Gu R., Liu Q., Chen S., Wang W., Wei X. (2019). Study on High-Temperature Mechanical Properties and Forming Limit Diagram of 7075 Aluminum Alloy Sheet in Hot Stamping. J. Mater. Eng. Perform..

[41] Wang A., Zhong K., El Fakir O., Liu J., Sun C., Wang L.-L., Lin J., Dean T.A. (2016). Springback analysis of AA5754 after hot stamping: Experiments and FE modelling. Int. J. Adv. Manuf. Technol..

[42] Zhang Z.-C., Xu Y.-C., Yuan S.-J. (2015). Analysis of thickness variation of reverse deep drawing of preformed 5A06 aluminum alloy cup under different temperatures. Int. J. Adv. Manuf. Technol..

[43] Bariani P.F., Bruschi S., Ghiotti A., Michieletto F. (2013). Hot stamping of AA5083 aluminium alloy sheets. CIRP Ann..

[44] Lian J., Shen F., Jia X., Ahn D.-C., Chae D.-C., Münstermann S., Bleck W. (2018). An evolving non-associated Hill48 plasticity model accounting for anisotropic hardening and r-value evolution and its application to forming limit prediction. Int. J. Solids Struct..

[45] Bruschi S., Ghiotti A., Michieletto F. (2013). Hot Tensile Behavior of Superplastic and Commercial AA5083 Sheets at High Temperature and Strain Rate. Key Eng. Mater..

[46] Ali R.O.A., Chatti S. (2019). Modeling springback of thick sandwich panel using RSM. Int. J. Adv. Manuf. Technol..

[47] Fan X.-B., He Z.-B., Zhou W.-X., Yuan S.-J. (2016). Formability and strengthening mechanism of solution treated Al–Mg–Si alloy sheet under hot stamping conditions. J. Mater. Process. Technol..

[48] Yang X., Zhang Q., Zheng Y., Liu X., Politis D., Fakir O.E., Wang L. (2021). Investigation of the friction coefficient evolution and lubricant breakdown behaviour of AA7075 aluminium alloy forming processes at elevated temperatures. Int. J. Extrem. Manuf..

[49] Liu Y., Zhu Z., Wang Z., Zhu B., Wang Y., Zhang Y. (2018). Flow and friction behaviors of 6061 aluminum alloy at elevated temperatures and hot stamping of a B-pillar. Int. J. Adv. Manuf. Technol..

